# Manganese Acts as an Environmental Inhibitor of Pseudomonas aeruginosa Biofilm Development by Inducing Dispersion and Modulating c-di-GMP and Exopolysaccharide Production via RbdA

**DOI:** 10.1128/jb.00003-23

**Published:** 2023-05-18

**Authors:** Soyoung Park, Jozef Dingemans, Karin Sauer

**Affiliations:** a Department of Biological Sciences, Binghamton University, Binghamton, New York, USA; b Binghamton Biofilm Research Center, Binghamton University, Binghamton, New York, USA; Geisel School of Medicine at Dartmouth

**Keywords:** *P. aeruginosa*, SagS, RbdA, manganese, biofilms, expolpoysaccharides, Pel, Psl, DipA, attachment, biofilm formation, biofilm matrix, dispersion, diverse environment, phosphodiesterases

## Abstract

The opportunistic human pathogen Pseudomonas aeruginosa causes chronic infections that involve multicellular aggregates called biofilms. Biofilm formation is modulated by the host environment and the presence of cues and/or signals, likely affecting the pool of the bacterial second messenger cyclic diguanylate monophosphate (c-di-GMP). The manganese ion Mn^2+^ is a divalent metal cation that is essential for pathogenic bacterial survival and replication during the infection in a host organism. In this study, we investigated how Mn^2+^ alters P. aeruginosa biofilm formation via the regulation of c-di-GMP levels. Exposure to Mn^2+^ was found to temporally enhance attachment but impair subsequent biofilm development, apparent by reduced biofilm biomass accumulation and lack of microcolony formation due to the induction of dispersion. Moreover, exposure to Mn^2+^ coincided with reduced production of the exopolysaccharides Psl and Pel, decreased transcriptional abundance of *pel* and *psl*, and decreased levels of c-di-GMP. To determine whether the effect of Mn^2+^ was linked to the activation of phosphodiesterases (PDEs), we screened several PDE mutants for Mn^2+^-dependent phenotypes (attachment and polysaccharide production) as well as PDE activity. The screen revealed that the PDE RbdA is activated by Mn^2+^ and is responsible for Mn^2+^-dependent attachment, inhibition of Psl production, and dispersion. Taken together, our findings suggest Mn^2+^ is an environmental inhibitor of P. aeruginosa biofilm development that acts through the PDE RbdA to modulate c-di-GMP levels, thereby impeding polysaccharide production and biofilm formation but enhancing dispersion.

**IMPORTANCE** While diverse environmental conditions such as the availability of metal ions have been shown to affect biofilm development, little is known about the mechanism. Here, we demonstrate that Mn^2+^ affects Pseudomonas aeruginosa biofilm development by stimulating phosphodiesterase RbdA activity to reduce the signaling molecule c-di-GMP levels, thereby hindering polysaccharide production and biofilm formation but enhancing dispersion. Our findings demonstrate that Mn^2+^ acts as an environmental inhibitor of P. aeruginosa biofilms, further suggesting manganese to be a promising new antibiofilm factor.

## INTRODUCTION

Bacteria can adapt to various surroundings by sensing environmental signals, resulting in altered gene expression and protein production, and/or changes in cellular processes, such as the mode of growth (1–5). Biofilms, multicellular communities encased in an extracellular polymeric matrix (EPS), are the dominant mode of growth in nature and during infections (6). The biofilm mode of growth is beneficial for bacteria as it allows cells to maintain close to nutrients, promotes the exchange of genetic material, and confers protection from a variety of chemical and environmental stresses (e.g., nutrient limitation, desiccation, and shear forces) (7). Moreover, biofilms are associated with persistent and chronic infections, including cystic fibrosis (CF), that are refractory to treatment by conventional antibiotics (8, 9). The opportunistic human pathogen Pseudomonas aeruginosa has been classified as one of the ESKAPE pathogens (i.e., Enterococcus faecium, Staphylococcus aureus, Klebsiella pneumoniae, Acinetobacter baumannii, *P. aeruginosa*, and *Enterobacter* species) that are highly recalcitrant to antibiotic treatment, and its biofilm formation is considered the primary cause of mortality in patients with CF (9, 10).

Biofilm formation by P. aeruginosa is a sequential and highly regulated process involving several distinct stages, including reversible and irreversible attachment, biofilm maturation stages I and II (defined by cluster and microcolony formation, respectively), and dispersion (11). The process is governed by several regulatory proteins, factors, and cues, including the surface sensing systems Pil-Chp and Wsp (12–14), SagS (15, 16), and environmental stimuli such as Psl polysaccharide (17, 18) or nitric oxide (NO) (19), leading to attachment and dispersion, respectively, as well as phosphodiesterases that contribute to dispersion, including NbdA (19, 20), RbdA, and DipA (19, 21). These various factors, cues, and/or signals have in common that they affect the intracellular level of the c-di-GMP that inversely regulates biofilm formation and motility (22–24).

Biofilm formation is furthermore affected by the presence and availability of metal ions. The effect of metal ion availability on biofilm formation has been investigated in several pathogenic bacteria, including P. aeruginosa. For example, magnesium limitation inhibits the expression of *retS* encoding the sensor kinase protein, which leads to increased EPS production and biofilm formation in P. aeruginosa (25). In contrast, levels of production of the biofilm matrix components CdrA and Psl are decreased in both mucoid and nonmucoid P. aeruginosa strains when grown under high-calcium conditions (26). The effect of iron on P. aeruginosa biofilm formation has also been explored. Elevated levels of iron reduce motility by P. aeruginosa cells and enhance biofilm microcolony formation, whereas iron-limiting conditions, obtained by the presence of the iron-binding glycoprotein lactoferrin, has been reported to coincide with reduced P. aeruginosa biofilm formation (27, 28). Exposure of P. aeruginosa to ferric citrate, which is transported actively into the cells, restored biofilm formation even in the absence of the iron scavenger pyoverdine, indicating that iron uptake is the determinant of biofilm formation (28). Moreover, under iron-rich condition, Psl is increased as a result of inhibition of *amrZ* expression and reduced rhamnolipid production, with Psl acting as an iron reservoir by sequestering iron (29). Iron affecting biofilm formation is consistent with sputum of CF patients harboring relatively high concentration of iron (398 to 1,292 μg/L) relative to healthy lungs (30).

Another metal ion, manganese (Mn^2+^), has been reported to be essential for pathogenic bacterial survival during the infection in a host organism as well as various cellular processes, such as metabolism, transcriptional regulation, and resistance to oxidative stress (31–33). Interestingly, however, Mn^2+^ concentrations in the sputum of CF patients have been shown to be relatively low, ranging from 4 to 17 μg/L or 0.1 to 0.8 μg/L (30, 34) and not differing significantly from the Mn^2+^ concentration present in the lungs of healthy patients or those suffering from chronic obstructive pulmonary disease (COPD) (34). Manganese has also been known to compete with iron for binding to enzymes such as Mn-superoxide dismutase due to similar sizes and coordination geometries with iron (31, 35). However, little is known about the role of Mn^2+^/manganese ions in P. aeruginosa biofilm development despite the structural similarity to iron and its physiological importance. The goal of this study was to determine the effect of manganese ions on P. aeruginosa biofilm formation and elucidate its mode of action.

## RESULTS

### Exposure of P. aeruginosa to Mn^2+^ enhances attachment.

**FIG 1:**
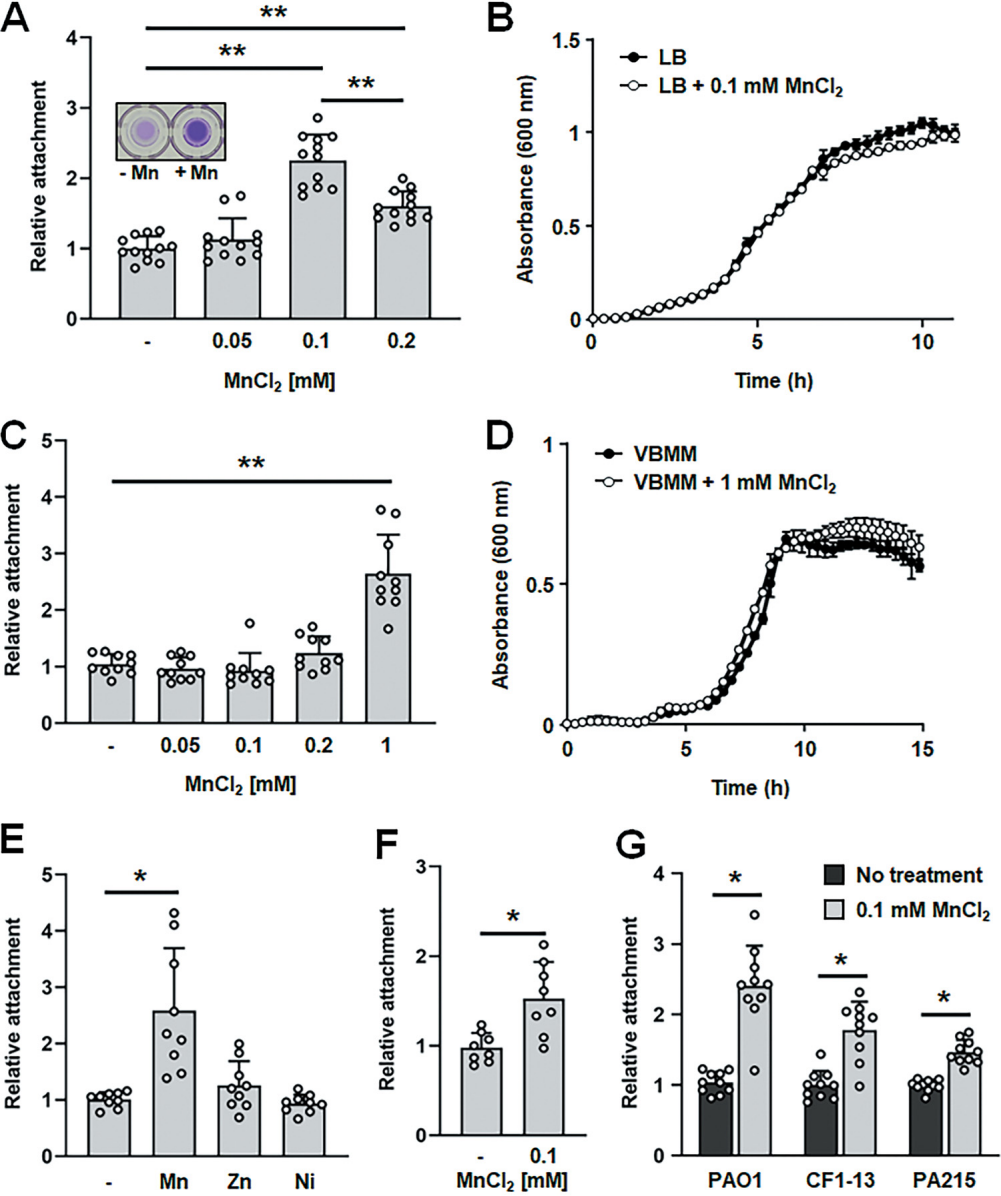
Effect of manganese ions on P. aeruginosa attachment. Attachment of P. aeruginosa PAO1 grown for 24 h in 96-well plates in LB (A and G), VBMM (C and E), or SDSU artificial sputum medium (ASM) (F). Attachment was assessed using crystal violet (CV) staining. Experiments were conducted in triplicate (two technical replicates each). Statistical significance relative to strain grown in the medium alone was assessed using one-way analysis of variance (ANOVA), followed by a Dunnett’s *post hoc* test. *, *P < *0.01; **, *P < *0.001. (A) Attachment of P. aeruginosa PAO1 grown in LB in the presence of 0 to 0.2 mM MnCl_2_. (B and D) Growth curve obtained for P. aeruginosa PAO1 supplemented with or without MnCl_2_ at the concentrations indicated. Absorbance was determined at 600 nm. The mean and standard deviation (SD) from four measurements are shown. P. aeruginosa was grown in (B) LB or (D) VBMM. (C) Attachment of P. aeruginosa PAO1 grown in VBMM in the presence of 0 to 1 mM MnCl_2_; (E) attachment by wild-type P. aeruginosa PAO1 grown in VBMM alone or supplemented with 1 mM MnCl_2_, ZnCl_2_, or NiCl_2_; (F) attachment of P. aeruginosa PAO1 grown in SDSU ASM in the presence of 0 and 0.1 mM MnCl_2_; (G) attachment of wild-type P. aeruginosa PAO1 and the clinical isolates CF1-13 and PA215 grown in LB in the presence or absence of 0.1 mM MnCl_2_.

Little is known about the effect of manganese on P. aeruginosa biofilm formation’s contribution to the chronicity of infections. To explore the role of manganese in the development of P. aeruginosa biofilms, we first examined whether manganese affects the attachment capabilities of P. aeruginosa. Attachment was assessed using 96-well plates and growing P. aeruginosa PAO1 in Lennox broth (LB) lacking or supplemented with manganese for 24 h. The attached biomass was quantitated by crystal violet (CV) staining. Cells grown in the presence of 0.1 and 0.2 mM MnCl_2_ showed increased CV-stainable biomass compared to its absence (Fig. 1A). However, elevated MnCl_2_ concentrations exceeding 0.1 mM MnCl_2_ (e.g., 0.2 mM) showed significant reduction in the CV-stainable biomass relative to that in attachment assays performed in the presence of 0.1 mM MnCl_2_ (Fig. 1A). To determine if the enhanced attachment was linked to enhanced growth, growth curve measurements in the absence and presence of 0.1 mM MnCl_2_ were performed. Under the conditions tested, no differences in the growth rates or growth behaviors were noted (Fig. 1B), suggesting that the increase in attachment by P. aeruginosa in LB supplemented with manganese was not the result of a general increase in growth rate.

To exclude the possibility that the complex LB may contain an unknown factor or factors involved in manganese-mediated attachment and determine whether the enhanced attachment was specific to manganese, we repeated the attachment assay using the defined Vogel and Bonner citrate minimal medium (VBMM) in the presence of increasing concentrations of MnCl_2_ (0.05, 0.1, 0.2, and 1 mM). In contrast to LB, addition of 0.1 and 0.2 mM MnCl_2_ did not coincide with a significant increase in attachment (Fig. 1C). However, addition of 1 mM MnCl_2_ significantly enhanced attachment by wild-type P. aeruginosa PAO1 in VBMM (Fig. 1C), suggesting that manganese-mediated increase in attachment is independent of the growth medium used. Moreover, no growth difference in VBMM supplemented with and without 1 mM MnCl_2_ was noted (Fig. 1D).

We next assessed relative attachment by wild-type P. aeruginosa PAO1 grown in VBMM supplemented with or without other divalent metal ions. Specifically, we tested ZnCl_2_ and NiCl_2_. However, no significant difference in attachment was noted (Fig. 1E), suggesting that manganese ions specifically, and not (divalent) metal ions in general, enhance attachment by P. aeruginosa.

We furthermore asked whether the manganese-dependent enhanced attachment is also apparent under conditions mimicking the host environment. Therefore, the attachment was assessed using the San Diego State University artificial sputum medium (SDSU ASM) in the presence/absence of 0.1 mM MnCl_2_. Similar to LB (Fig. 1A), the presence of 0.1 mM MnCl_2_ in SDSU medium coincided with increased attachment (Fig. 1F). The finding suggested that manganese ions also enhance attachment under conditions mimicking *in vivo* conditions. We furthermore explored whether the response was limited to the P. aeruginosa PAO1 laboratory strain. We therefore selected two P. aeruginosa clinical strains, CF1-13 and PA215, that were previously isolated from the CF lung and burn wounds, respectively. Their attachment capabilities were determined using LB supplemented with or without 0.1 mM MnCl_2_. Like P. aeruginosa PAO1 (Fig. 1A), the clinical isolates exhibited enhanced attachment capabilities in the presence of manganese relative its absence (Fig. 1G), suggesting that this effect is not limited to the laboratory strain.

### Exposure of P. aeruginosa to Mn^2+^ reduces biofilm biomass accumulation and the formation of structured biofilms.

**FIG 2:**
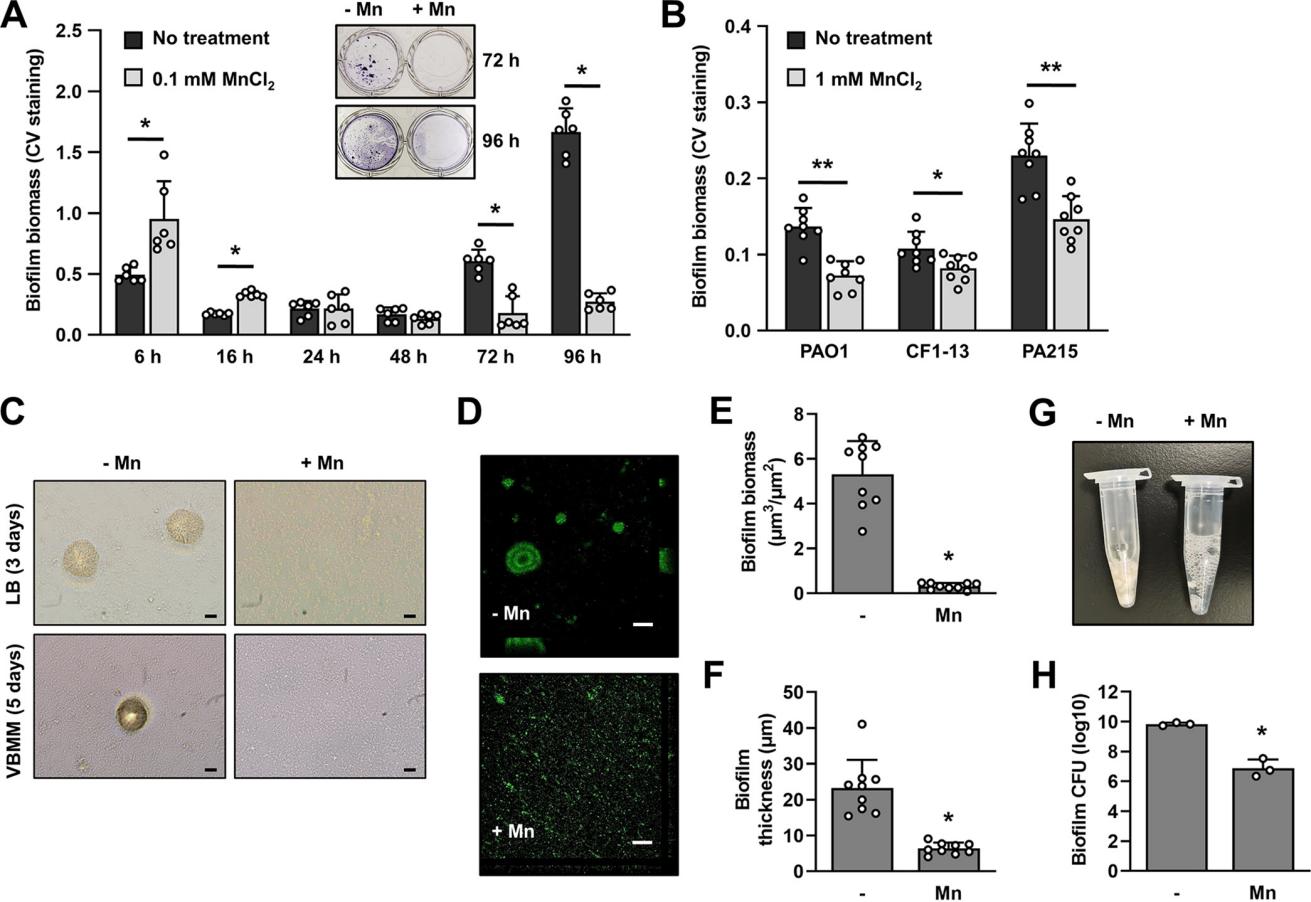
Effect of manganese ions on P. aeruginosa biofilm formation. (A to F) P. aeruginosa biofilms were grown in 24-well polystyrene plates in 5-fold diluted LB or VBMM supplemented with 0, 0.1, or 1 mM MnCl_2_. (A) The level of biofilm biomass by 5-fold-diluted LB-grown P. aeruginosa PAO1 at different time points was determined by CV staining. Experiments were conducted in triplicate using two technical replicates. Statistical significance was assessed using Student's *t* test. *, *P < *0.01. (B) Level of biofilm biomass of P. aeruginosa PAO1 and the clinical isolates CF1-13 and PA215 grown for 5 days in 5-fold diluted VBMM medium supplemented with or without 1 mM MnCl_2_, as determined using CV staining. Experiments were performed in triplicate using at least two technical replicates. Statistical significance was assessed using Student's *t* test. **, *P < *0.01; *, *P < *0.05. (C) Representative bright-field microscopy images of P. aeruginosa PAO1 biofilms grown for 3 or 5 days in either 5-fold-diluted LB or VBMM in the presence or absence of Mn^2+^. Bars, 20 μm. (D) Representative confocal microscopy images demonstrating the architecture of P. aeruginosa PAO1 biofilms grown for 3 days in 5-fold-diluted LB in the presence or absence of Mn^2+^. Biofilms were stained prior to microscopy using a LIVE/DEAD BacLight viability stain. Bars, 100 μm. The total biofilm biomass (E) and biofilm thickness (F) were determined by COMSTAT analysis. *, statistically different (*P < *0.0001) by unpaired, two-tailed *t* test relative to biofilms grown without Mn^2+^. (F and G) P. aeruginosa biofilms were grown for 3 days in tube reactors in 20-fold-diluted LB in the absence or presence of 0.1 mM MnCl_2_. (F) Representative image of biomass accumulation of the biofilms; (G) average number of viable cells present in the biofilms as determined using CFU count. *, significantly different (*P* < 0.01) by Student's *t* test relative to biofilms grown in the absence of manganese. Shown are the mean and standard deviation (*n *= 3 independent measurements).

Since the exposure of P. aeruginosa to manganese enhanced attachment, we assumed that manganese also stimulates subsequent biofilm formation. To verify this assumption, we grew P. aeruginosa biofilms in 24-well plates in 5-fold-diluted LB supplemented with or without 0.1 mM MnCl_2_ for up to 96 h and assessed the accumulation of the biofilm biomass by CV staining at 6, 16, 24, 48, 72, and 96 h postinoculation. As expected, exposure to manganese coincided with a significantly high biomass accumulation of P. aeruginosa PAO1 at early time points (6 and 16 h) relative to LB alone (Fig. 2A). However, manganese enhancement of biomass accumulation was temporary, as continued incubation in the presence of Mn^2+^ resulted in significantly reduced biofilm biomass, with the inhibitory effect first noticeable following 3 days of biofilm growth (Fig. 2A). A similar inhibitory effect on biofilm biomass was noted for 5-day-old biofilms formed by P. aeruginosa PAO1 and the two clinical isolates CF1-13 and PA215 when grown in 5-fold-diluted VBMM supplemented with 1 mM MnCl_2_ (Fig. 2B).

Our observations indicated that Mn^2+^ enhanced attachment, but reduced subsequent biofilm biomass accumulation upon continued exposure. This prompted us to explore how Mn^2+^ affects the development of P. aeruginosa biofilms. Daily monitoring over a period of 5 days by bright-field microscopy indicated that P. aeruginosa forms structured biofilms composed of microcolonies within 3 and 5 days in 5-fold-diluted LB and VBMM, respectively (Fig. 2C). In contrast, P. aeruginosa grown in the presence of Mn^2+^ failed to develop the typical wild-type biofilm architecture (Fig. 2C), indicating that exposure to Mn^2+^ impairs the formation of structured biofilms. Exposure to Mn^2+^ affecting the biofilm architecture was confirmed by confocal microscopy, apparent by untreated, 5-fold-diluted LB-grown 3-day-old biofilms featuring a structured biofilm architecture composed of large microcolonies ~100 μm in diameter, while Mn^2+^-treated biofilms appeared to be thin and unstructured (Fig. 2D). COMSTAT analysis confirmed biofilms grown in the presence of manganese ions to be composed of 3 to 5 times less biomass and reduced biofilm height relative to the untreated biofilms (Fig. 2E and F).

The microscopic analysis combined with COMSTAT suggested exposure to Mn^2+^ not only impairs microcolony formation but also affects the overall biofilm biomass. To confirm that exposure to Mn^2+^ indeed affects the overall biofilm biomass accumulation, biofilms were grown under flowing conditions in tube reactors in the presence or absence of Mn^2+^, and the resulting biofilm biomass was harvested after 3 days of biofilm growth. Visible differences in the biofilm biomass were noted (Fig. 2G), with viability counts confirming exposure to Mn^2+^ coinciding with an overall reduction in the CFU/biofilm relative to untreated biofilms (Fig. 2H).

### Manganese affects attachment and biofilm formation in a manner dependent on SagS.

**FIG 3:**
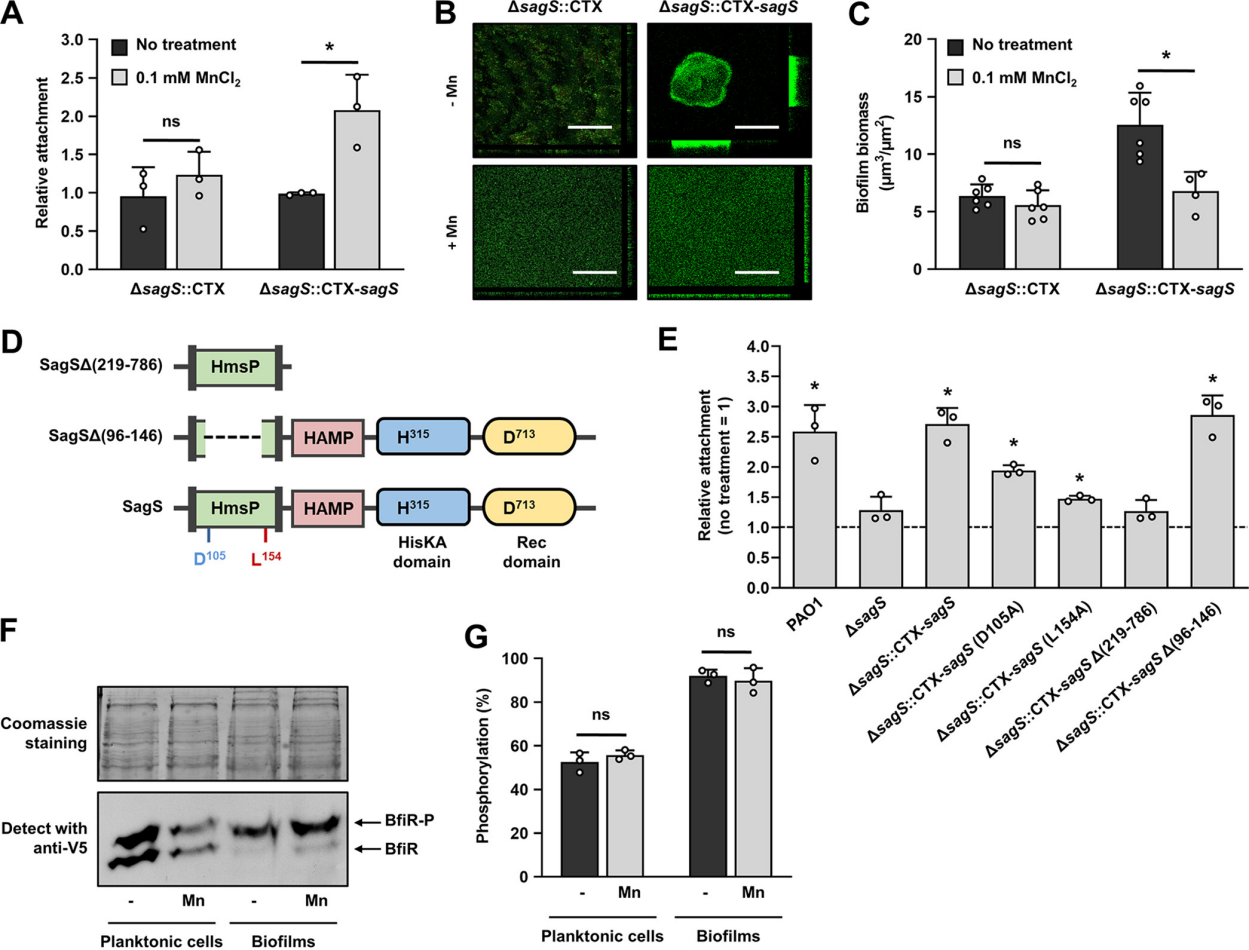
Manganese is not perceived by SagS. (A) Attachment of LB-grown Δ*sagS*::CTX and Δ*sagS*::CTX-*sagS* strains after 24 h of growth in 96-well plates in the presence or absence of 0.1 mM MnCl_2_ as determined using CV staining. Statistical significance relative to strain grown in LB alone was assessed using Student's *t* test (*, *P < *0.01). Shown are the mean and standard deviation (*n *= 3 independent measurements). (B) Representative confocal microscopy images of the biofilm architecture by Δ*sagS*::CTX and Δ*sagS*::CTX-*sagS* cells. Biofilms were grown for 3 days in 24-well plates in 5-fold-diluted LB supplemented with or without 0.1 mM MnCl_2_ and stained prior to microscopy using a LIVE/DEAD BacLight viability stain. Bars, 100 μm. (C) Quantification of the total biomass as determined by COMSTAT analysis. *, significantly different (*P < *0.05) by Student's *t* test compared to untreated biofilms. (D) Schematic presentation of the wild-type and a truncated version of SagS constructed to test the attachment capability. The wild-type or truncation mutations of *sagS* were integrated into the PAO1 Δ*sagS* chromosome at the *attB* site. H^315^ and D^713^ are SagS conserved phosphorelay sites, and D^105^ and L^154^ are residues associated with antibiotic tolerance and biofilm formation, respectively. (E) Effect of manganese on attachment. Attachment capabilities of each strain were determined by CV staining assay following 24 h of growth in 96-well plates in LB supplemented with or without 0.1 mM MnCl_2_. *, significantly different (*P* < 0.001) by Student's *t*-test from each strain tested in the absence of manganese. Shown are the mean and standard deviation (*n *= 3 biological replicates). (F) Phos-tag Western blot analysis of cell extracts prepared from wild-type P. aeruginosa harboring a plasmid expressing the BfiR-V5 grown supplemented with or without manganese in either the planktonic or biofilm mode of growth. Biofilms were grown for 3 days in tube reactors, and planktonic cells were grown to exponential phase. Coomassie-stained SDS gels showing total cell extracts after transfer were used as loading controls. Data are representative of three independent experiments, which produced similar results. (G) Determination of the percentage of phosphorylated BfiR over total BfiR, based on the Phos-tag Western blot analysis. Band intensities were analyzed using ImageJ software. Experiments were performed using biological triplicates. ns, not significant by Student's *t* test.

The effect of Mn^2+^ on attachment and biofilm formation was reminiscent of the role of SagS in biofilm development, with the inactivation of *sagS* coinciding with enhanced attachment but the formation of thin and unstructured biofilms (15). We therefore asked whether SagS is required for Mn^2+^ to affect both attachment and biofilm formation. We reasoned that if SagS is required, the inactivation of *sagS* would render the effect of Mn^2+^ negligible. To address this question, we made use of a Δ*sagS* mutant (Δ*sagS*::CTX) and an *sagS* complemented strain (Δ*sagS*::CTX-*sagS*). Similar to P. aeruginosa PAO1, exposure of the Δ*sagS*::CTX-*sagS* strain to Mn^2+^ significantly enhanced attachment, while the same treatment had little to no effect on Δ*sagS*::CTX (Fig. 3A). Likewise, exposure to Mn^2+^ failed to affect the biomass accumulation or architecture of biofilms formed by Δ*sagS*::CTX relative to untreated mutant biofilms, but significantly reduced the biofilm biomass accumulation and architecture of biofilms formed by the complemented *sagS* mutant strain (Fig. 3A and B). Our visual observations by confocal microscopy were confirmed by the quantitative analysis of the biofilm biomass by COMSTAT (Fig. 3C). Our findings suggested Mn^2+^ affects attachment and biofilm formation in a manner dependent on SagS.

### Manganese is not a cue for SagS.

In order to elucidate whether SagS contributed to Mn^2+^-dependent attachment and biofilm formation via sensing Mn^2+^, we investigated the role of SagS domains and amino acid residues previously reported to contribute to SagS sensory function (16, 36, 37) (Fig. 3D). SagS harbors three domains, including an N-terminally located HmsP sensory domain, a centrally located histidine kinase (HisKA) domain and a C-terminal receiver (Rec) domain (Fig. 3D). We generated truncated SagS variants that either (i) comprised only the periplasmic HmsP sensory domain or (ii) lacked the sensory HmsP domain, with both SagS variants retaining the transmembrane helices, ensuring proper localization in the inner membrane (Fig. 3D). In addition, SagS variants harboring amino acid substitutions D105A and L154A were investigated (Fig. 3D). The amino acid residue L154 has been reported to be important in biofilm development (37), while residue D105 has been linked to biofilm antibiotic tolerance but not biofilm development (37). Δ*sagS* mutant strains harboring the respective SagS variant constructs were subsequently evaluated for their response to manganese using 96-well plate attachment assays (Fig. 3E). P. aeruginosa PAO1 and the complemented *sagS* strain were used as positive controls. With the exception of the SagS variant composed of only the periplasmic HmsP sensory domain, variants resulted in the Δ*sagS* mutant, demonstrating enhanced attachment in the presence relative to the absence of Mn^2+^ (Fig. 3E). Our findings suggested Mn^2+^ was not perceived by the periplasmic sensory domain of SagS. SagS has been known to promote biofilm formation through hierarchical phosphotransfer to the two-component system BfiSR (15). To further explore the role of SagS, we determined whether exposure to Mn^2+^ affects the phosphorylation status of BfiR. We therefore made use of P. aeruginosa harboring expressing V5-tagged *bfiR* grown under planktonic and biofilm growth conditions in the absence and presence of Mn^2+^ and evaluated the phosphorylation status of BfiR by immunoblot analysis using Phos-tag acrylamide. In agreement with previous findings (38), BfiR was found to be more phosphorylated under biofilm than under planktonic growth conditions (Fig. 3F and G). However, no difference in the BfiR phosphorylation state was noted in the absence or presence of Mn^2+^ (Fig. 3F and G), further confirming SagS is not activated by or does not perceive Mn^2+^.

### Exposure to Mn^2+^ affects c-di-GMP levels.

**FIG 4:**
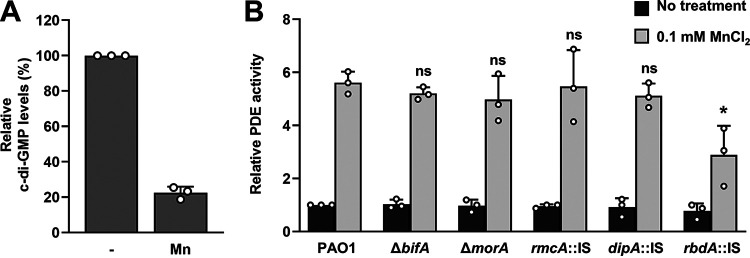
Manganese ions affect c-di-GMP levels via the PDE RbdA. (A) Relative levels of intracellular c-di-GMP in P. aeruginosa PAO1 cells grown to a late stationary phase, harboring the unstable c-di-GMP reporter P*cdrA*::gfp(ASV) and pmCherry. The relative fluorescence (RFU) was normalized to mCherry-based fluorescence. *, significantly different (*P < *0.01) by Student’s *t* test relative to cells grown in the absence of Mn^2+^. Shown are the mean and the standard deviation (*n *= 3 independent replicates). (B) Relative phosphodiesterase (PDE) activity. Wild-type and indicated mutant strains were grown planktonically to the late stationary phase in LB alone or supplemented with 0.1 mM MnCl_2_. PDE activity assays were carried out using the chromogenic substrate bis-pNPP and 100 μg total cell extracts. *, significantly different (*P < *0.001) by one-way ANOVA, followed by a Dunnett’s *post hoc* test, relative to PAO1 grown in LB supplemented with Mn^2+^; ns, not significant. Shown are the mean and standard deviation (*n *= 3 independent measurements).

The second messenger c-di-GMP has emerged as a key modulator of the lifestyle transition between motility and sessility (22–24). High intracellular levels of c-di-GMP enhance biofilm formation, whereas its low levels promote a motile lifestyle, leading to biofilm dispersion and returning to a planktonic state (22–24). To further elucidate the mechanism by which Mn^2+^ temporally enhances attachment, but impairs the formation of structured biofilms, we next asked whether Mn^2+^affects the pool of c-di-GMP. We, therefore, made use of the P*_cdrA_*::gfp(ASV) reporter, for which the fluorescence intensity is directly proportional to the intracellular c-di-GMP concentration (39). As expected, when wild-type cells were grown planktonically to the late stationary phase in the presence of Mn^2+^, green fluorescent protein (GFP) fluorescence significantly decreased compared to in its absence, indicating that Mn^2+^ reduces c-di-GMP levels (Fig. 4A). The findings suggested Mn^2+^ is capable of affecting c-di-GMP production.

### Mn^2+^ stimulate overall PDE activity by P. aeruginosa PAO1.

The cellular pool of c-di-GMP is controlled by two classes of enzymes with opposing activities, diguanylate cyclases (DGCs) and phosphodiesterases (PDEs) (22, 23), with reduced cellular levels of c-di-GMP likely being due to attenuated DGC activity and/or increased PDE activity (22, 23). We first assessed whether exposure of P. aeruginosa cells to Mn^2+^ has an overall effect on the PDE activity. We therefore prepared total cell extracts of P. aeruginosa PAO1 cells grown planktonically to the late stationary phase in LB alone or supplemented with Mn^2+^. The resulting cell extracts were subsequently used to determine the overall PDE activity assays using the synthetic PDE substrate bis(*p*-nitrophenyl) phosphate (bis-pNPP) (Fig. 4B). Relative to cell extracts obtained from cells grown in LB alone, addition of Mn^2+^ to the growth medium coincided with a 5.6-fold increase in the PDE activity by P. aeruginosa PAO1, suggesting that exogenous Mn^2+^ indeed stimulates the PDE activity, with the increase in PDE activity likely leading to reduced c-di-GMP levels (Fig. 4A and B).

We next sought to identify the Mn^2+^-responsive PDE. We reasoned that inactivation of such a PDE would coincide with a reduction in PDE activity in the presence of Mn^2+^ to levels comparable to those in the absence of Mn^2+^. However, if the PDE contributes to reduced c-di-GMP levels in an Mn^2+^-independent manner, the respective mutant strain would show similar PDE activity to wild-type cells in the presence of Mn^2+^. We focused on several PDEs known to contribute to early biofilm formation stages and/or the overall pool of c-di-GMP in biofilms. These included BifA, RmcA, MorA, RbdA, and DipA. BifA has been reported to contribute to biofilm formation in early stages, by inhibiting the synthesis of c-di-GMP and Pel in P. aeruginosa PA14 (40), while the PDEs RmcA and MorA have been reported to contribute to the maintenance of the mature biofilm structure in response to nutrient limitations (41). Moreover, RbdA and DipA have been shown to contribute to the formation of the mature biofilm architecture, not only by contributing to the overall pool of c-di-GMP but also by promoting dispersion and, thus, the return to a planktonic mode of growth by reducing intracellular c-di-GMP levels (19, 21). Therefore, we made use of two P. aeruginosa mutants, the Δ*bifA* and Δ*morA* strains, harboring in-frame deletions of *bifA* and *morA*, respectively, and mutants harboring transposon insertion elements (ISs) in *rmcA*, *dipA*, or *rbdA*. The latter mutants are referred to as the *rmcA*::IS, *dipA*::IS, and *rbdA*::IS strains. PDE activity assays were performed using total cell extracts obtained from PDE mutants grown planktonically to the late stationary phase in the presence or absence of 0.1 mM MnCl_2_. Under the conditions tested, the PDE activity by P. aeruginosa PAO1 in response to Mn^2+^ increased ~6-fold (Fig. 4B). Similarly, the PDE activity in cell extracts obtained from Δ*bifA*, Δ*morA*, *rmcA*::IS and *dipA*::IS mutants increased on average 5.6-fold in response to Mn^2+^ (Fig. 4B). In contrast, however, a much reduced response to Mn^2+^ was noted by the *rbdA* mutant (*rbdA*::IS) strain (Fig. 4B). While the PDE activity was somewhat increased relative to the *rbdA* mutant grown in the absence of Mn^2+^, the PDE activity increased on average only 2.5-fold (Fig. 4B). The findings strongly suggested the PDE activity of RbdA is enhanced by Mn^2+^.

### Mn^2+^ stimulates the PDE activity of RbdA.

**FIG 5:**
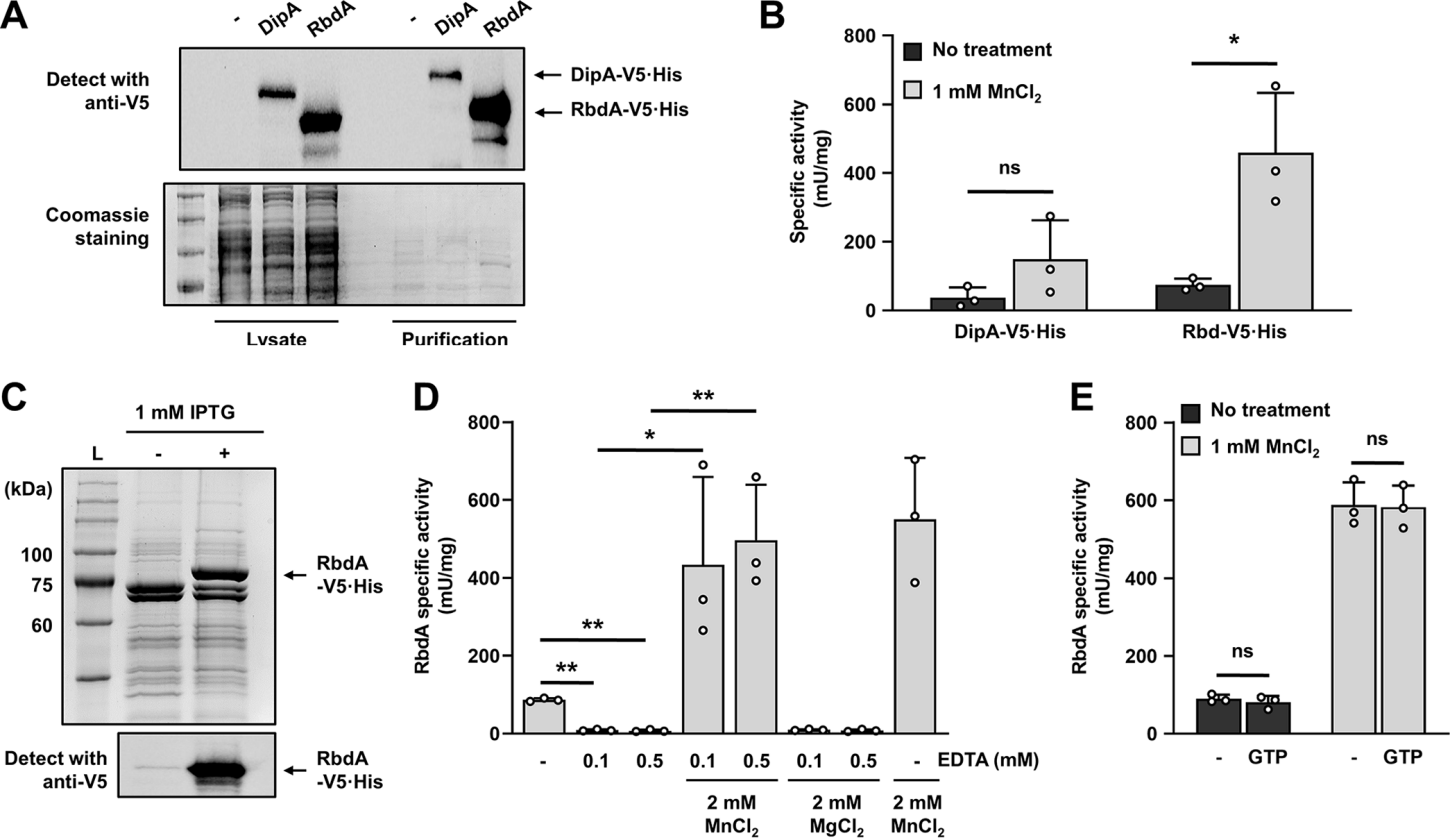
Manganese ions stimulate the PDE activity of RbdA. (A) Evaluation of stability and purification of DipA-V5/His and RbdA-V5/His. The Western blot shows the levels of DipA and RbdA, either in cell extracts of P. aeruginosa PAO1 harboring the plasmid pMJT-*dipA* or pMJT-*rbdA* or in Ni-NTA eluates obtained after protein purifications. P. aeruginosa PAO1 harboring pMJT-1 was used as a negative control. Proteins were detected using an anti-V5 antibody. Coomassie-stained SDS-PAGE gels after transfer were used as loading controls. (B) Specific PDE activity of purified proteins in the absence or presence of Mn^2+^. *, significantly different (*P < *0.05) by unpaired, two-tailed *t* test in the presence relative to the absence of Mn^2+^; ns, not significant. (C) V5-His tagged RbdA was purified from E. coli BL21 grown in the absence and presence of IPTG and analyzed by SDS-PAGE, followed by Coomassie staining or Western blotting with an anti-V5-antibody. Lane L, ladder of molecular weight markers (GeneTex). (D) Effect of Mn^2+^ and Mg^2+^ on the PDE activity of RbdA. Divalent cations were removed from RbdA by treatment with 0.1 or 0.5 mM EDTA for 10 min before the protein was reconstituted with Mg^2+^ or Mn^2+^. EDTA-untreated RbdA proteins with or without Mn^2+^ were used as controls. Asterisks indicate statistically significant difference between the indicated conditions (*, *P < *0.05; **, *P < *0.01) by Student's *t* test. (E) Effect of GTP on the PDE activity of RbdA in the presence and absence of Mn^2+^. The PDE activity of RbdA (1. 98 μM) was assessed in the presence and absence of GTP (5 μM). Statistical significance was determined by an unpaired, two-tailed *t* test. ns, not significant. (B, D, and E) bis-pNPP was used as an artificial substrate of PDE assays, with the resulting *p*-nitrophenol being measured at 405 nm. Shown are means and standard deviations from *n *= 3 independent measurements.

PDEs have been reported to use a two-metal mode of catalysis, with likely metal ions including Mn^2+^, Co^2+^, Ni^2+^, and Mg^2+^ (42). To determine whether the PDE activity of RbdA is indeed responsive to Mn^2+^, we next purified C-terminally V5/6×His-tagged RbdA from P. aeruginosa wild-type cells (Fig. 5A). Purified C-terminally V5/6× His-tagged DipA was used as a negative control (Fig. 5A). Purified enzymes were subsequently subjected to PDE activity assays. In the absence of Mn^2+^, purified enzymes harbored an average specific activity of 40 to 75 mU/mg (Fig. 5B). No significant increase in the activity of DipA was noted upon addition of 1 mM MnCl_2_ (Fig. 5B). In contrast, the PDE activity of RbdA increased ~6-fold to a specific activity of ~460 mU upon addition of 1 mM MnCl_2_ (Fig. 5B).

To ensure that the activity of RbdA is indeed enhanced by addition of 1 mM MnCl_2_, we subjected RbdA to chelating conditions to remove divalent ions, followed by add-back of specific metal ions. To do so, purified RbdA (purified from Escherichia coli [Fig. 5C]) was exposed for 10 min to EDTA (0.1 and 0.5 mM) to remove divalent ions. Following chelation, the enzymatic activity of RbdA in the presence of EDTA in the absence or presence of 2 mM MnCl_2_ was evaluated (Fig. 5D). Untreated RbdA in the absence of MnCl_2_ served as a positive control (Fig. 5D). Under the conditions tested, untreated RbdA in the absence of MnCl_2_ had a specific PDE activity of 87 mU/mg, which increased 6.3-fold to 550 mU/mg in the presence of MnCl_2_ (Fig. 5D). Pretreatment of RbdA for 10 min with 0.1 and 0.5 mM EDTA resulted in a significant, 10-fold reduction in the specific activity relative to untreated RbdA (Fig. 5D). Addition of 2 mM MnCl_2_ almost fully restored the PDE activity of EDTA-treated RbdA to that noted for untreated RbdA in the presence of Mn^2+^ (Fig. 5D). It is of interest to note that the addition of 2 mM MgCl_2_ failed to restore the RbdA activity posttreatment with EDTA (Fig. 5D). Our findings strongly suggested that not only is the PDE RbdA responsible for the overall increase in PDE activity upon exposure to Mn^2+^, but the activity of RbdA is also significantly enhanced by Mn^2+^. Our findings are in agreement with previous reports of Mn^2+^ enhancing PDE activity (42). However, while Mn^2+^ and Mg^2+^ can frequently be exchanged without loss of enzyme activity, our findings clearly indicate that Mn^2+^ cannot be substituted by Mg^2+^ to activate RbdA.

RbdA is composed of an N-terminal PAS-PAC domain, a GGDEF (possessing DGC activity) domain, and a C-terminal EAL (possessing PDE activity) domain (43). The PDE activity of RbdA has been reported to be stimulated when GTP binds to its GGDEF domain (43, 44). We therefore asked whether GTP would affect the Mn^2+^-stimulated PDE activity of RbdA. To explore the GTP effect, we examined the PDE activity of purified RbdA (purified by E. coli [Fig. 5C]) by adding an excess amount of GTP in the absence and presence of Mn^2+^. Addition of GTP, however, did not significantly enhance the PDE activity of RbdA, regardless of the absence and presence of Mn^2+^ (Fig. 5E).

### RbdA contributes to the Mn^2+^ dependency of biofilm formation.

**FIG 6:**
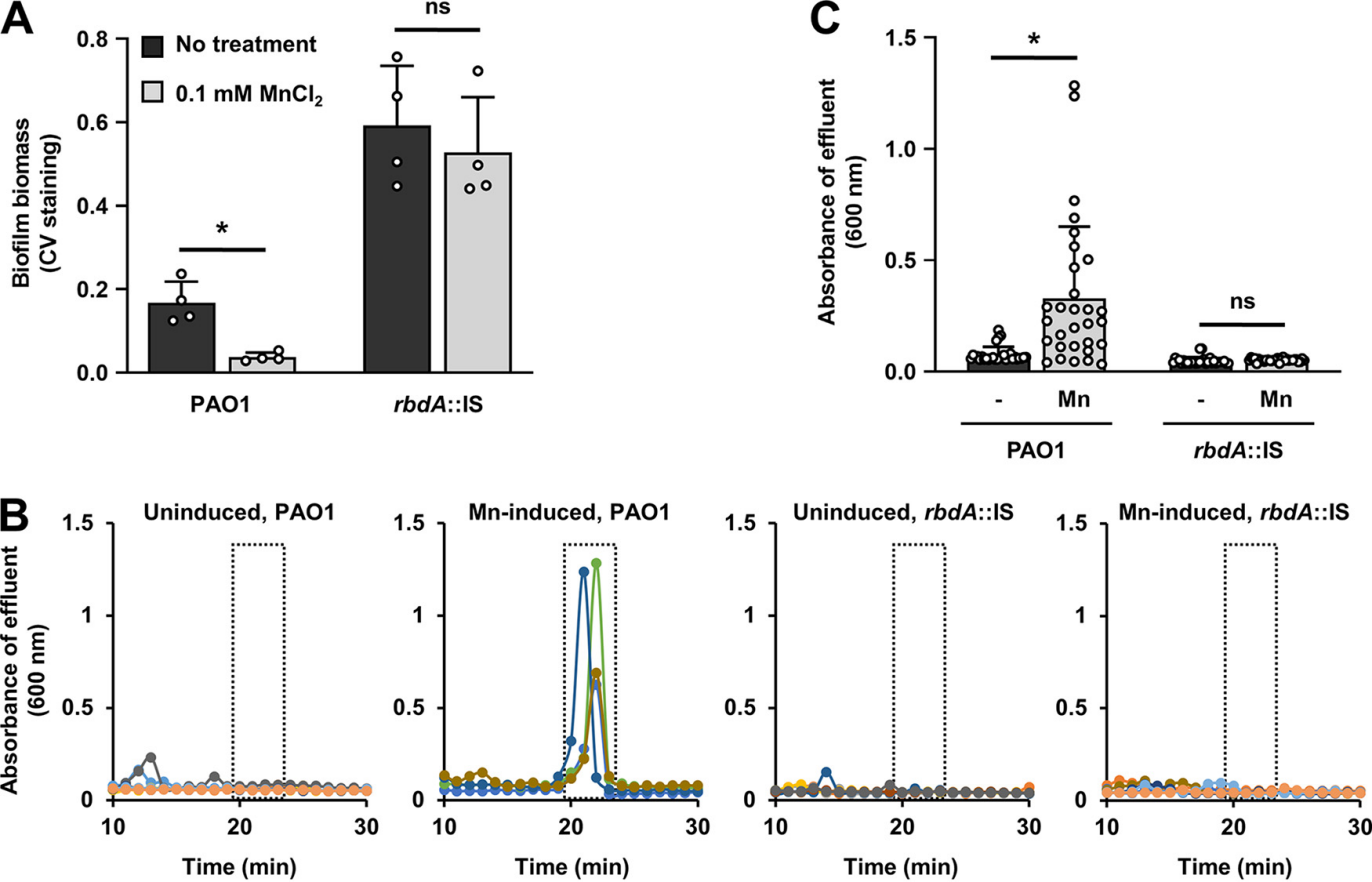
Manganese contributes to biofilm development via RbdA. (A) Biofilms were grown for 3 days in 24-well polystyrene plates in 5-fold-diluted LB alone or supplemented with 0.1 mM MnCl_2_. The level of biofilm biomass was determined by CV staining. Statistical significance (*, *P < *0.01) was determined by Student's *t* test. ns, not significant. Shown are the mean and standard deviation from *n *= 4 independent measurements. (B) Dispersion of biofilms by P. aeruginosa wild-type or the *rbdA*::IS mutant strain upon exposure to 0.5 mM MnCl_2_ or medium alone. Biofilms were grown for 5 days in 5-fold-diluted VBMM in tube reactors. The absorbance of biofilm tube reactor effluents after induction of dispersion is shown. Dispersion assays were performed in triplicate using at least three technical replicates. Representative dispersion profiles are shown. (C) The absorbance of effluents collected 20 to 23 min (see dashed boxes in panel B) after exposure to 0.5 mM MnCl_2_ or medium alone (untreated) was selected and quantified. *, significantly different (*P < *0.001) by unpaired, two-tailed *t* test relative to untreated biofilms; ns, not significant.

Our findings indicated RbdA to be responsive to Mn^2+^. We therefore reasoned that if manganese ions indeed affect biofilm formation by stimulating the PDE activity of RbdA, and thus, the overall level of c-di-GMP, inactivation of *rbdA* would eliminate or at least significantly diminish the effect of Mn^2+^ on biofilm formation. To examine the role of RbdA in the biofilm formation in response to Mn^2+^, we grew biofilms formed by the wild-type and *rbdA*-deficient mutant strains in 24-well plates for 3 days in the presence or absence of Mn^2+^ and determined the level of biofilm biomass accumulations by CV staining. While the biofilm biomass by the P. aeruginosa wild-type strain was significantly reduced in the presence of Mn^2+^ relative to its absence, no significant difference in the CV-stainable biomass was noted in biofilms formed by the *rbdA*::IS mutant strain (Fig. 6A). The findings indicated that the overall reduction in the biofilm biomass in response to Mn^2+^ is due to RbdA and its responsiveness to Mn^2+^.

### RbdA contributes to biofilm dispersion in response to Mn^2+^.

RbdA has previously been reported to be contribute to biofilm dispersions in response to exogenous dispersion cues such as glutamate and nitric oxide (19, 21). Given that Mn^2+^ contributes to the loss of biofilm biomass and that RbdA is responsive to Mn^2+^, we next asked if exposure to Mn^2+^ induces biofilm dispersion in a manner dependent on RbdA. To address this question, we performed a dispersion assay using MnCl_2_ as a cue to induce dispersion. Biofilms formed by the P. aeruginosa wild-type or *rbdA*::IS mutant strain were grown under flow conditions in a tube reactor for 5 days and subsequently exposed to a sudden change of growth medium supplemented with 0.5 mM MnCl_2_. Biofilm effluents were collected after exposure to MnCl_2_-containing medium, and the absorbance of the effluents was subsequently determined at 600 nm. Dispersion events have previously been reported to be apparent by a sharp increase in the absorbance in the effluent upon induction of dispersion compared to untreated biofilms (20, 21, 45, 46).

Exposure of wild-type biofilms to growth medium supplemented with 0.5 mM MnCl_2_ resulted in a sharp increase in the absorbance of the biofilm effluents, indicative of dispersion events, a response that was absent in biofilms that were left untreated and exposed to growth medium alone (Fig. 6B and C). However, biofilms formed by the *rbdA*::IS mutant strain failed to disperse in response to Mn^2+^ (Fig. 6B and C). It is of interest to note that biofilms formed by *rbdA*::IS mutant have been reported to be comparable to wild type in architecture but demonstrating enhanced biofilm biomass accumulation (43). The finding strongly suggested Mn^2+^ to be a likely cue capable of inducing biofilm dispersion, with Mn^2+^-induced dispersion to be the likely cause for the significant loss of biomass of biofilms grown in the presence of Mn^2+^ (Fig. 2 and Fig. 6A). Moreover, our data indicated an RbdA dependency of both dispersion and loss of biofilm biomass in response to Mn^2+^ (Fig. 6).

### Mn^2+^ affects expression of *pel* and *psl* genes and abundance of Pel and Psl polysaccharides.

**FIG 7:**
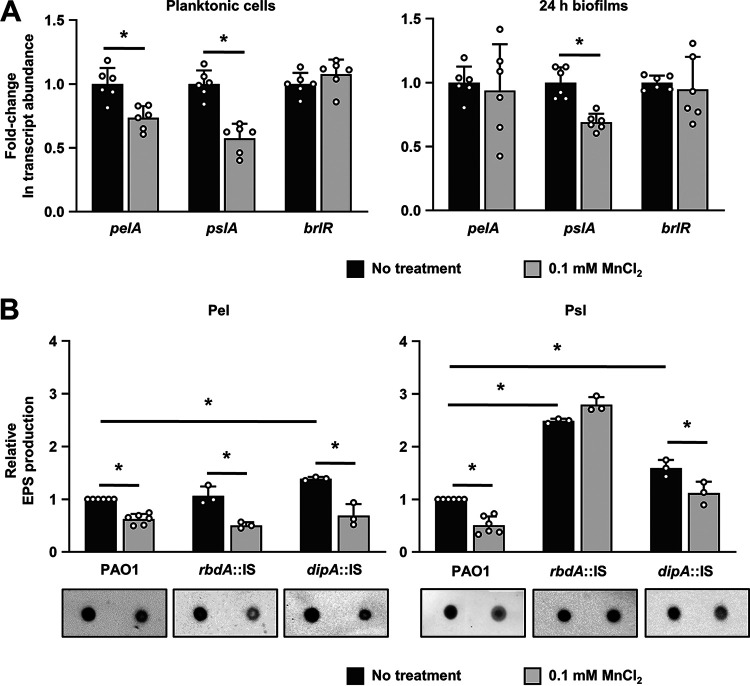
Psl production is dependent on the PDE RbdA. (A) Fold change in expression levels of the *pelA*, *pslG*, and *brlR* genes in the absence and presence of 0.1 mM MnCl_2_ by qRT-PCR in cells grown planktonically to the late stationary phase and as biofilms (attached for 24 h). The transcript abundance was normalized to the expression of P. aeruginosa PAO1 in the absence of Mn^2+^. *cysD* was used as a housekeeping gene. Experiments were conducted in triplicate using two technical replicates each. *, significantly different (*P < *0.01) by Student's *t* test relative to untreated controls. (B) Pel (left) and Psl (right) polysaccharide abundance relative to P. aeruginosa PAO1 in the presence/absence of Mn^2+^. The abundance of Pel and Psl was assessed using planktonic cells grown to the late stationary phase in LB alone or supplemented with 0.1 mM MnCl_2_ and anti-Pel or anti-Psl dot blot analysis. The asterisk indicates statistical significance (*, *P < *0.01) by Student's *t* test. Shown are means and standard deviations from *n *= 6 independent experiments for P. aeruginosa PAO1 and *n *= 3 independent experiments for mutants.

We next explored the mechanism by which RbdA affects biofilm biomass in a Mn^2+^-dependent manner. We took into account the finding that the second messenger c-di-GMP has been reported to stimulate Psl and Pel production and to be elevated by Psl exopolysaccharide, establishing a positive-feedback loop (22, 24). Moreover, previous reports indicated that Pel and Psl exopolysaccharides are degraded upon dispersion (46) and that matrix exopolysaccharides, including alginate, Pel, and Psl, affect attachment and microcolony formation (47–49). Specifically, lack of Psl has been reported to coincide with significantly reduced initial attachment (47). In addition, while both Psl and alginate are required for the formation of characteristic mushroom-like structures (47–49), Pel appeared to play a role in biofilm cell density and/or the compactness of the biofilm (47–49). In contrast, mutants deficient in both Psl and Pel production (but capable of producing alginate) were severely impaired in their ability to form biofilms (48). As our findings indicated exposure to Mn^2+^ to coincide with reduced intracellular c-di-GMP levels via stimulation of RbdA activity (Fig. 4 and Fig. 5) and the formation of thin biofilms lacking microcolonies or cellular aggregates (Fig. 2), we asked whether Mn^2+^ affects the expression of genes involved in the biosynthesis (and degradation) of the polysaccharides Pel and Psl. We therefore determined the transcript abundance of *pelA* and *pslG*, parts of *pel* and *psl* operons, respectively, by quantitative reverse transcription-PCR (qRT-PCR). PelA, encoded by *pelA*, is a periplasmic modification enzyme with an N-terminal glycoside hydrolase domain and a C-terminal deacetylase domain (50), whereas PslG, encoded by *pslG*, has been characterized to harbor Psl glycoside hydrolase activity (51). Under planktonic growth conditions, the transcript abundance of *pelA* and *pslG* was significantly reduced upon exposure to Mn^2+^ (Fig. 7A). Likewise, biofilms exposed to Mn^2+^ for 24 h demonstrated significantly decreased transcript abundance of *pslG*, but not *pelA*, relative to untreated biofilms (Fig. 7A). It is noteworthy that, under the conditions tested, the expression level of *brlR*, encoding the biofilm resistance locus regulator, showed no significant differences regardless of the absence or presence of Mn^2+^ (Fig. 7A). To determine whether differential expression of *pelA* and *pslG* transcript in response to Mn^2+^ correlated with differences in the abundance of Pel and Psl polysaccharides, the respective polysaccharides were purified from P. aeruginosa PAO1 grown planktonically to the exponential phase in the presence or absence of Mn^2+^. The Pel and Psl polysaccharides were subsequently detected by immunoblot analysis using anti-Pel and anti-Psl antibodies, respectively (Fig. 7B). In agreement with our qRT-PCR analysis, the quantitative analysis of Pel and Psl abundance indicated exposure to Mn^2+^ coincides with the reduced abundance of the Psl and Pel polysaccharides (Fig. 7B).

### Psl abundance is dependent on RbdA.

RbdA has been known to negatively regulate exopolysaccharide production (43). Considering that our findings so far suggested Mn^2+^ affects the expression of *pel* and *psl* as well as the abundance of the Pel and Psl exopolysaccharides, we next asked whether Mn^2+^-mediated inhibition of Pel and Psl requires RbdA.

Relative to P. aeruginosa PAO1, insertional inactivation of *rbdA* had no effect on Pel abundance (Fig. 7B). In contrast, insertional inactivation of *dipA* correlated with an up to 1.5-fold increase in Pel abundance relative to the wild type (Fig. 7B). Exposure of the *rbdA*::IS and *dipA*::IS strains to Mn^2+^ resulted in a reduction of Pel abundance in a manner comparable to the overall reduction in Pel abundance by wild-type cells (Fig. 7B). Insertional inactivation of *dipA* and *rbdA* affected the overall abundance of Psl, with the abundance of Psl being increased 2- and 2.5-fold in *dipA*- and *rbdA*-deficient mutant strains, respectively, relative to the wild type (Fig. 7B). Similar to Pel, the abundance of Psl decreased in the *dipA*-deficient mutant strains in response to Mn^2+^ in a manner similar to that of the wild-type strain (Fig. 7B). In contrast, however, no difference in Psl abundance was noted in the *rbdA*-deficient mutant strain regardless of the absence or presence of Mn^2+^ (Fig. 7B), suggesting RbdA contributes to the abundance of the Psl response to Mn^2+^. Taken together, our data demonstrate that Mn^2+^ acts through the PDE RbdA to impede not only Psl production but also the formation of structured biofilms.

## DISCUSSION

Several studies have explored the effects of Mn^2+^ on biofilm formation. A combination of Mn^2+^ and glycerol has been found to promote biofilm formation of *Bacillus* species such as *B. subtilis*, B. licheniformis, and B. cereus via the histidine kinase KinD (52). Hussain et al. (53), demonstrated that the expression levels of genes involved in motility and EPS formation were positively correlated with B. cereus biofilm formation in brain heart infusion (BHI) medium supplemented with Mn^2+^ and heme. The same medium induced B. cereus biofilm formation (53). However, Guo et al. (54) showed that Mn^2+^ inhibits E. coli biofilm formation in a concentration-dependent manner through the regulation of phenotypic morphology and metabolic reprogramming (54). It has also been shown that exposure of P. putida MnB1 to Mn^2+^ for 12 h coincided with an upregulation of *bifA*, accompanied by biofilm suppression (55). While the studies indicated Mn^2+^ to be a potential cue to modulate biofilm formation, no mechanism has been elucidated. The goal of this study was to determine whether Mn^2+^ affects biofilm formation by P. aeruginosa and, if so, elucidate the mechanism by which Mn^2+^ modulates P. aeruginosa biofilm formation. Here, we showed that Mn^2+^ initially enhances attachment (Fig. 1) but subsequently inhibits P. aeruginosa biofilm formation (Fig. 2) by reducing intracellular c-di-GMP levels (Fig. 4A) and Psl polysaccharide abundance (Fig. 7). Our findings of Mn^2+^ reciprocally affecting attachment and biofilm formation (as well as inducing dispersion) provide an explanation for the inconsistency of the role of Mn^2+^ in the literature. Our mechanistic study revealed Mn^2+^ alters P. aeruginosa biofilm development via modulation of the second messenger c-di-GMP. Specifically, we demonstrate that Mn^2+^ acts through the PDE RbdA to reduce c-di-GMP levels (Fig. 4 and 5), thereby reducing Psl abundance and biofilm formation (Fig. 6 and 7).

Our findings are furthermore in agreement with reports by An et al. (43) characterizing RbdA as an active PDE capable of affecting the production of exopolymeric substances, determined by Congo red staining and pellicle formation assays. However, despite providing several lines of evidence demonstrating a link between EPS production and RbdA activity, An et al. (43) did not provide direct evidence of EPS abundance being affected by RbdA activity nor specified the EPS (Pel, Psl, or alginate). In this study, we show that RbdA contributes to the abundance of the exopolysaccharide Psl, not Pel (Fig. 7). To our knowledge, this is the first report of Psl production being dependent on RbdA.

Enzymatic activities, especially PDE activities, being stimulated by metal ions are not unusual. For example, the EAL domain from the c-di-GMP specific-PDE HmsP of Yersinia pestis exhibited high PDE activity in the presence of 1 mM manganese relative to the presence of other divalent metals such as Zn and Mg, indicating that the PDE activity depends on Mn^2+^ (56). Similarly, the metallophosphoesterase PlcP by *Phaeobacter* sp. strain MED193 and *Pelagibacter* sp. strain HTCC7211 is essential for lipid remodeling, with its phosphomonoesterase and phosphodiesterase activity being dependent on manganese (57). The pGpG-specific PDE PggH of Vibrio cholerae showed the highest PDE-B activity in the presence of Mn^2+^ even though the enzyme also showed activity in the presence of divalent cations such as Mg^2+^, Ca^2+^, and Zn^2+^ (58). In contrast, the divalent cations Mg^2+^ and Mn^2+^ were necessary for V. cholerae VieA activity, while Ca^2+^ and Zn^2+^ inhibited the PDE activity of VieA (59). Other c-di-GMP-modulating enzymes are magnesium dependent. For instance, the c-di-GMP-specific PDE activities of CC3399, a GGDEF-EAL protein from Caulobacter crescentus, and RocR, an EAL domain protein from P. aeruginosa PAO1, require Mg^2+^ (60, 61). Our findings of EDTA-treated RbdA being restored in its activity by Mn^2+^ but not Mg^2+^ (Fig. 5D) indicate that RbdA is a manganese-inducible PDE. However, the mechanism by which manganese affects the PDE activity of RbdA remains to be elucidated. Liu et al. (44) suggested that a signal detected by the N-terminal putative periplasmic sensor domain of RbdA or GTP binding to the GGDEF domain may lead to a conformational change and, thus, RbdA dimerization and PDE activity activation. It is thus likely that manganese may be such a signal, either inducing conformational change of RbdA to activate its PDE function or modulating the PDE activity by binding to one of its domains (e.g., GGDEF).

Our findings clearly indicated that exposure of mature biofilms formed by P. aeruginosa to Mn^2+^ coincided with reduced biofilm biomass accumulation, reduced microcolony formation, and induction of dispersion via the modulation of c-di-GMP and that the effect by Mn^2+^ is dependent on RbdA, leading to the modulation in Psl abundance. As the Psl polysaccharide has been reported to protect P. aeruginosa from host defenses in an acute murine pulmonary model of infection (62), our findings underscore the possibility that Mn^2+^ may be able to modulate the P. aeruginosa virulence repertoire via Psl. Therefore, our study demonstrates that Mn^2+^ acts as an environmental inhibitor of P. aeruginosa biofilm development, suggesting Mn^2+^ to be a promising new antibiofilm factor.

## MATERIALS AND METHODS

### Bacterial strains, plasmids, and culture conditions.

The bacterial strains, plasmids, and oligonucleotides used in this study are listed in Table 1 and Table 2. The complex medium, Lennox Broth (LB), was used as a growth medium for P. aeruginosa and E. coli. The minimal medium, Vogel and Bonner citrate minimal medium (VBMM) (63), and the artificial sputum medium (ASM), SDSU (64, 65), were used to grow P. aeruginosa PAO1 and the clinical isolates. All planktonic cells were grown at 37°C under shaking conditions (220 rpm). Biofilms were grown as indicated below. The following antibiotics were added for plasmid maintenance: for P. aeruginosa, 60 μg mL^−1^ tetracycline, 75 μg mL^−1^ gentamicin, and 250 μg mL^−1^ carbenicillin; for E. coli, 15 μg mL^−1^ tetracycline, 20 μg mL^−1^ gentamicin, and 100 μg mL^−1^ ampicillin. Additionally, 1 mM isopropyl-β-d-1-thiogalactopyranoside (IPTG) or 0.1% l-arabinose was added to the growth medium where indicated.

**TABLE 1:** Strains and plasmids used in this study

Strain or plasmid	Genotype and/or description^*a*^	Source or reference
Strains		
P. aeruginosa		
PAO1	Wild-type PAO1	B. H. Holloway
Δ*sagS*	PAO1 Δ*sagS* (PA2824)	43
Δ*sagS*::CTX	Δ*sagS* strain harboring empty pMini CTX vector	68
Δ*sagS*::CTX-*sagS*	Δ*sagS* strain harboring chromosomal insertion of *sagS* under control of *sagS* promoter at *attB* site; cured pMini CTX vector	68
Δ*sagS*::CTX-*sagS*_D105A	Δ*sagS* strain harboring chromosomal insertion of *sagS*_D105A under control of *sagS* promoter at *attB* site; cured pMini CTX vector	68
Δ*sagS*::CTX-*sagS*_L154A	Δ*sagS* strain harboring chromosomal insertion of *sagS*_L154A under control of *sagS* promoter at *attB* site; cured pMini CTX vector	68
Δ*sagS*::CTX-*sagS* Δ(219–786)-HA	Δ*sagS* strain harboring chromosomal insertion of *sagS* Δ(219–786) under control of *sagS* promoter at *attB* site; Tet^r^	This study
Δ*sagS*::CTX-*sagS* Δ(96–146)-HA	Δ*sagS* strain harboring chromosomal insertion of *sagS* Δ(96–146) under control of *sagS* promoter at *attB* site; Tet^r^	This study
Δ*bifA*	PAO1 Δ*bifA* (PA4367)	Gift from T. Tolker-Nielsen (69)
Δ*morA*	PAO1 Δ*morA* (PA4601)	This study
*rmcA*::IS	PAO1 PA0575::IS*lacZ*; Tet^r^	70
*dipA*::IS	PAO1 PA5017::IS*lacZ*; Tet^r^	70
*rbdA*::IS	PAO1 PA0861::IS*lacZ*; Tet^r^	70
CF1-13	Mucoid P. aeruginosa isolate from newborn diagnosed with CF	71
PA215	P. aeruginosa isolated from chronic wound debridement samples from patients at Southwest Regional Wound Clinic (Lubbock, TX)	72
E. coli		
DH5α	F^−^ ϕ80*lacZ*ΔM15 Δ(*lacZYA*-*argF*)*U169 recA1 endA1 hsdR17*(r_K_^−^ m_K_^+^) *phoA supE44 thi-1 gyrA96 relA1 tonA*	New England Biolabs
SM10 λ*pir*	*thi-1 thr leu tonA lacY supE recA*::RP4-2-Tc::Mu λ*pir*; OriT of RP4; Km^r^; conjugational donor	73
BL21	F^−^ *ompT gal dcm lon hsdS*_B_ (r_B_^−^ m_B_^−^) λ(DE3)	Invitrogen
Plasmids		
pRK2013	Helper plasmid for triparental mating; *mob tra* Km^r^	74
pET101D	Vector for directional cloning and high-level V5/6×His fusion protein expression; Amp^r^	Invitrogen
pMJT-1	*araC*-P_BAD_ cassette of pJN105 cloned into pUCP18; Amp^r^ (Carb^r^)	75
pMJT-*nicD*-V5/6×His	C-terminal V5/6×His-tagged *nicD* cloned into pMJT1 at NheI/XbaI; Amp^r^ (Carb^r^)	45
pET-*rbdA*-V5/6×His	*rbdA* cloned into pET101D; Amp^r^	Lab stock
pMJT-*rbdA-*V5/6×His	C-terminal V5/6×His-tagged *rbdA* cloned into pMJT1 at NheI/XbaI; Amp^r^ (Carb^r^)	76
pMJT-*dipA-*V5/6×His	C-terminal V5/6×His-tagged *dipA* cloned into pMJT1 at NheI/XbaI; Amp^r^ (Carb^r^)	21
pKO PA14_60870	PA14_60870 (*morA*) knockout construct in suicide vector pMQ30; Gm^r^	Gift from G. A. O’Toole (41)
pCdrA::*gfp*(ASV)	pUCP22Not-P_cdrA_-RBS-CDS-RNase III-*gfp*(ASV)-*T*_0_-*T*_1_ Amp^r^ Gm^r^	39
pMF440	Broad-host-range plasmid for constitutive expression of mCherry; Amp^r^ (Carb^r^)	Michael Franklin (Addgene plasmid no. 62550)

a Tet^r^, tetracycline resistant; Gm^r^, gentamicin resistant; Amp^r^, ampicillin-resistant; Carb^r^, carbenicillin resistant.

**TABLE 2:** Oligonucleotides used in this study

Name	Oligonucleotide sequence (5′→3′)	Use(s)
pMJT1 MCS_F	GACCGCGAATGGTGAG	PCR/sequencing
pMJT1 MCS_R	GAGCTGATACCGCTCG	
GFP-89F	GTCAGTGGAGAGGGTGAAGG	
GFP-538R	CTGCTAGTTGAACGCTTCCATC	
mCherry-F	GCGCTTCAAGGTGCACATGGAGGGC	
mCherry-R	CTTGTACAGCTCGTCCATGCCGCCG	
*pelA*-F	GGTGCTGGAGGACTTCATC	qRT-PCR
*pelA*-R	GGATGGCTGAAGGTATGGC	
*pslG*-F	CACGTAAGGGACTCTATCTGG	
*pslG*-R	CGGTCGATCTGCTTGTTGTAAC	
*brlR*-F	CAGCGTGGTGGGCATGGAATACTT	
*brlR*-R	AAGCCGGCGACGTAGTGGAATTC	
*cysD*-F	CTGGACATCTGGCAATACAT	
*cysD*-R	TCTCTTCGTCAGAGAGATGC	
*sagS* Δ(219–786)-F	GCCGGCACGCTACCCATACGACGTCCCAGACTACGCTTAG	Cloning
*sagS* Δ(219–786)-R	GTATGGGTAGCGTGCCGGCGAGCGTGGATCGTGTCCG	
*sagS* Δ(96–146)-F	CTGCGCTCGCCGGGCGAGGCCCTCGGCGTACTGCAC	
*sagS* Δ(96–146)-R	CTCGCCCGGCGAGCGCAGCAGGCCGAGGACCAGCTCCTG	

### Biofilm growth.

P. aeruginosa biofilms were grown in 20-fold-diluted LB or 5-fold-diluted VBMM using a continuous-flow tube reactor system with a size of 13 for biofilm biomass accumulation for 3 days and 14 Masterflex silicone tubing for dispersion assays for 5 days (Cole Parmer, Inc.) at flow rates of 0.1 and 0.2 mL min^−1^, respectively (11). Tube reactors were inoculated with 1 mL of P. aeruginosa overnight cultures. For a 24-well plate biofilm cultivation, biofilms were grown in 5-fold-diluted LB or VBMM, as previously described with some modifications (66). Briefly, overnight cultures of P. aeruginosa were adjusted to an optical density at 600 nm (OD_600_) of 0.1, and 10 μL of the OD-adjusted suspension was transferred into wells of 24-well flat-bottom polystyrene microplates (BD Falcon), with each well containing 250 μL medium. (The wells can hold a total volume of 1 mL but only 250 μL medium was added to prevent spills and cross-contamination.) The plate was incubated at 37°C and 220 rpm at a 45° angle. The 45° angle promotes biofilm formation by increasing the air-liquid interface. The medium was exchanged every 12 h. Adherent cells were quantitated using crystal violet (CV) staining. Briefly, 300 μL 0.1% CV solution was directly added to each well, followed by incubation for 15 min at 37°C with shaking. The plate was washed three times with 500 μL of water and then allowed to dry prior to the addition of 500 μL of 95% ethanol to each well. After incubation for 15 min at 37°C with shaking, the OD_570_ was determined. Quantitative analysis of the confocal laser scanning microscopy (CSLM) images of 24-well plate-grown biofilms, acquired by a Leica TCS SP5 (Leica Microsystems, Inc.), was performed using COMSTAT (5, 15). For confocal image acquisition, biofilms were stained with the LIVE/DEAD BacLight bacterial viability kit (Life Technologies). For plasmid maintenance, the growth medium was supplemented with carbenicillin (10 μg mL^−1^) or gentamicin (2 μg mL^−1^).

### Attachment assays.

Overnight-grown P. aeruginosa cells were washed using 0.85% saline and inoculated into LB, VBMM, or SDSU medium supplemented with or without MnCl_2_, ZnCl_2_, or NiCl_2_ at a final OD_600_ of 0.05. Then, 100 μL of the suspension was transferred into a 96-well plate and incubated with shaking at 220 rpm at 37°C for 24 h. The wells were subsequently rinsed with 0.85% saline to remove planktonic cells, and the remaining adherent cells were stained with 0.1% CV solution for 15 min. After being washed with 200 μL of water two times, the remaining crystal violet was solubilized with 200 μL of 95% ethanol and the absorbance measured at 570 nm.

### Biofilm dispersion assays.

Biofilm dispersion assays were performed using biofilms grown in continuous-flow tube reactors as previously described (45, 46). In brief, the dispersion of 5-day-old biofilms was induced by the sudden addition of 0.5 mM MnCl_2_ to the growth medium. Then, the biofilm effluents were collected into 96-well plates at 1-min intervals and the optical density if the collected effluent measured at 600 nm. An increase in the turbidity of the effluent indicated dispersion events. For the quantitative analysis of the dispersion response, the absorbance of effluents collected 20 to 23 min after exposure to 0.5 mM MnCl_2_ and that of untreated biofilms were evaluated.

### Determination of the phosphorylation state of BfiR.

Planktonic and 3-day old biofilm cells were cultured as described above and normalized by measuring the OD_600_. The cell pellets were resuspended in 45 μL of buffer A (50 mM Tris-HCl [pH 7.5], 150 mM NaCl, 1 mM EDTA) supplemented with 0.2 mg/mL lysozyme and 0.1 mg/mL DNase I, followed by repeated pipetting up and down for 10 s. Lysates were diluted into SDS loading buffer and analyzed by SDS-PAGE gels (12%) supplemented with 50 μM Phos-tag acrylamide (Wako) and 100 μM MnCl_2_ as described by the manufacturer. Electrophoresis was performed at 4°C and 120 V for 3 h. Before immunoblotting, the Phos-tag gel was incubated for 10 min in transfer buffer containing 10 mM EDTA and for another 10 min in transfer buffer twice to remove EDTA. Subsequent immunoblotting was performed using anti-V5-horseradish peroxidase (HRP) antibody (1:5,000) (Invitrogen). The blots were developed with Immun-Star WesternC chemiluminescent reagents (Bio-Rad). The densities of protein bands were determined by quantification using ImageJ software version 1.48 (NIH).

### Quantification of c-di-GMP.

Quantification of relative-c-di-GMP levels was performed using cells harboring plasmids expressing GFP(ASV) from the c-di-GMP-responsive *cdrA* promoter [pCdrA::gfp(ASV)] and expressing mCherry from a constitutive promoter as previously described, with some modifications (5, 39). P. aeruginosa cells biofilms grown for 3 days in LB supplemented with or without 0.1 mM MnCl_2_ were harvested, resuspended into 200 μL phosphate-buffered saline (PBS), and the suspension was transferred into a 96-well black clear-bottom microtiter plate (Greiner Bio-One). The fluorescent emission of GFP (excitation 485 nm/emission 535 nm) and mCherry (580 nm/620 nm) was measured every 30 min using a SpectraMax i3x plate reader (Molecular Devices). Fluorescence units from GFP were normalized to mCherry.

### Phosphodiesterase activity assay.

Phosphodiesterase activity was determined using the synthetic substrate bis(*p*-nitrophenyl) phosphate (bis-pNPP) (Sigma-Aldrich), as previously described (21, 40). Briefly, either 100 μg of crude extract or 18 μg of purified protein was incubated with 5 mM bis-pNPP in reaction buffer (50 mM Tris-HCl [pH 8.0], 50 mM NaCl, and 5 mM MgCl_2_) at 25°C for 4 h. The release of *p*-nitrophenol (pNP) was quantified at OD_405_ every 30 min. Crude extracts were obtained from cells grown planktonically in the absence of presence of MnCl_2._ Controls without extracts or purified protein were included to account for any nonenzymatic bis-pNPP hydrolysis.

### Purification of His-tagged proteins.

For protein purification, cells were grown planktonically to the exponential phase. Proteins harboring C-terminal V5/His tags cloned into pET vectors (E. coli) and pMJT-1 vectors (P. aeruginosa) were overproduced using 1 mM IPTG and 0.1% l-arabinose, respectively, and purified using Ni-nitrilotriacetic acid (NTA) metal-affinity resin (Thermo Fisher Scientific) according to the manufacturer’s instruction. Briefly, harvested cells were resuspended in lysis buffer (50 mM Tris-HCl [pH 7.5], 150 mM NaCl, 1 mM EDTA, 0.2 mM phenylmethylsulfonyl fluoride), lysed by sonication, and centrifuged at 10,000 × *g* at 4°C for 10 min to remove cell debris. After removal of cell debris from the lysate, membrane-bound proteins were solubilized by 1% (wt/vol) *n*-dodecyl-β-d-maltoside (DDM) and loaded onto Ni-NTA metal-affinity resin. Proteins bound to the resin were eluted with 200 mM imidazole and then desalted using VivaSpin centrifugal concentrator columns and a Tris buffer (50 mM Tris-HCl [pH 7.5], 100 mM NaCl, 5% glycerol) containing 0.05% DDM.

### RNA isolation and qRT-PCR.

Total RNA was isolated from cells grown planktonically and as a 3-day biofilm using a E.Z.N.A. Total RNA kit (Omega Bio-Tek), and DNA was removed using Turbo DNase (Thermo Fisher Scientific) as previously described (5). The same amount of RNA (1 μg) from each cell was converted to cDNA using the iScript Select cDNA synthesis kit (Bio-Rad). qRT-PCR was carried out using the Bio-Rad CFX Connect real-time PCR detection system and SsoAdvanced SYBR green supermix (Bio-Rad). Specific primers for amplification of the cDNA are listed in Table 2. To normalize the transcript level, the *cysD* gene was used as a reference.

### Pel and Psl polysaccharide dot blot analysis.

Crude polysaccharide extracts were obtained by resuspending 10 OD_600_ equivalents of cells from each growth condition in 100 μL of 0.5 M EDTA and boiling for 10 min at 100°C as described previously (67). The supernatant was treated with proteinase K at 60°C for 1 h (final concentration 0.5 mg mL^−1^), followed by proteinase K inactivation at 80°C for 30 min. For immunoblotting, 5 μL of polysaccharide extract was spotted on a nitrocellulose membrane and probed as described previously (21, 67). The Pel and Psl productions were quantified using ImageJ software version 1.48 (NIH).
